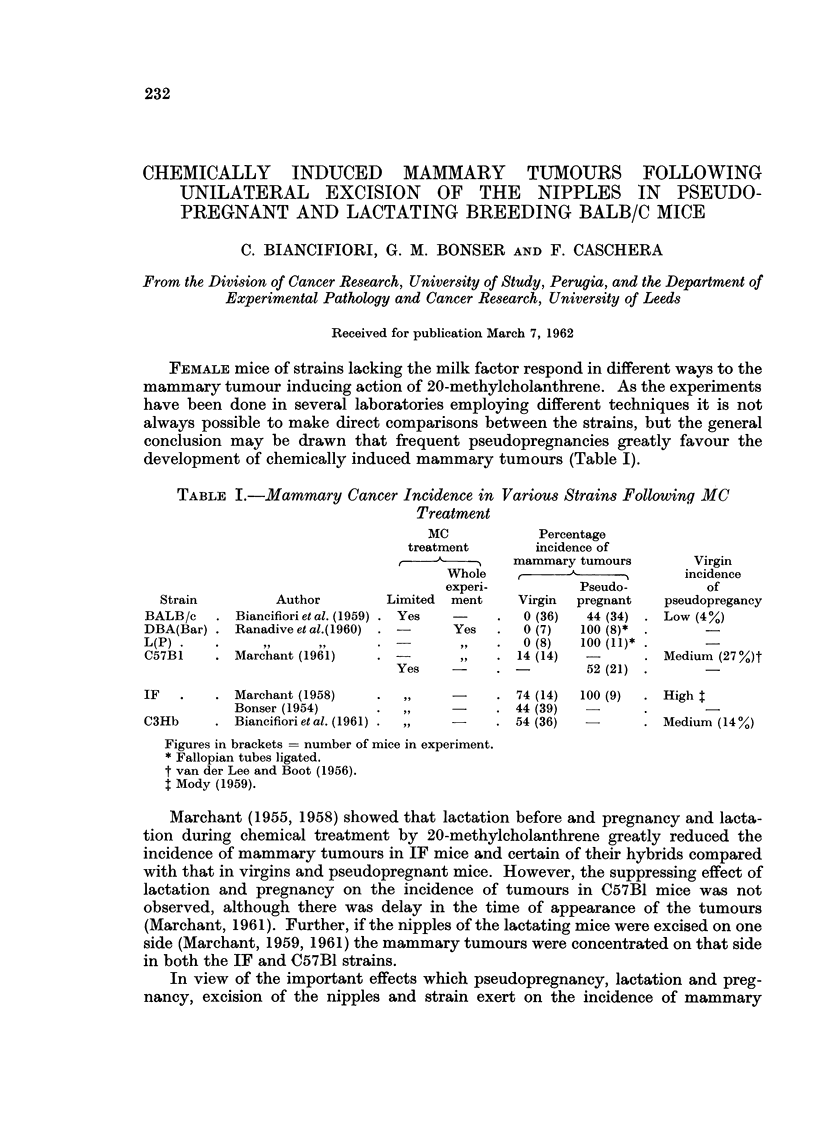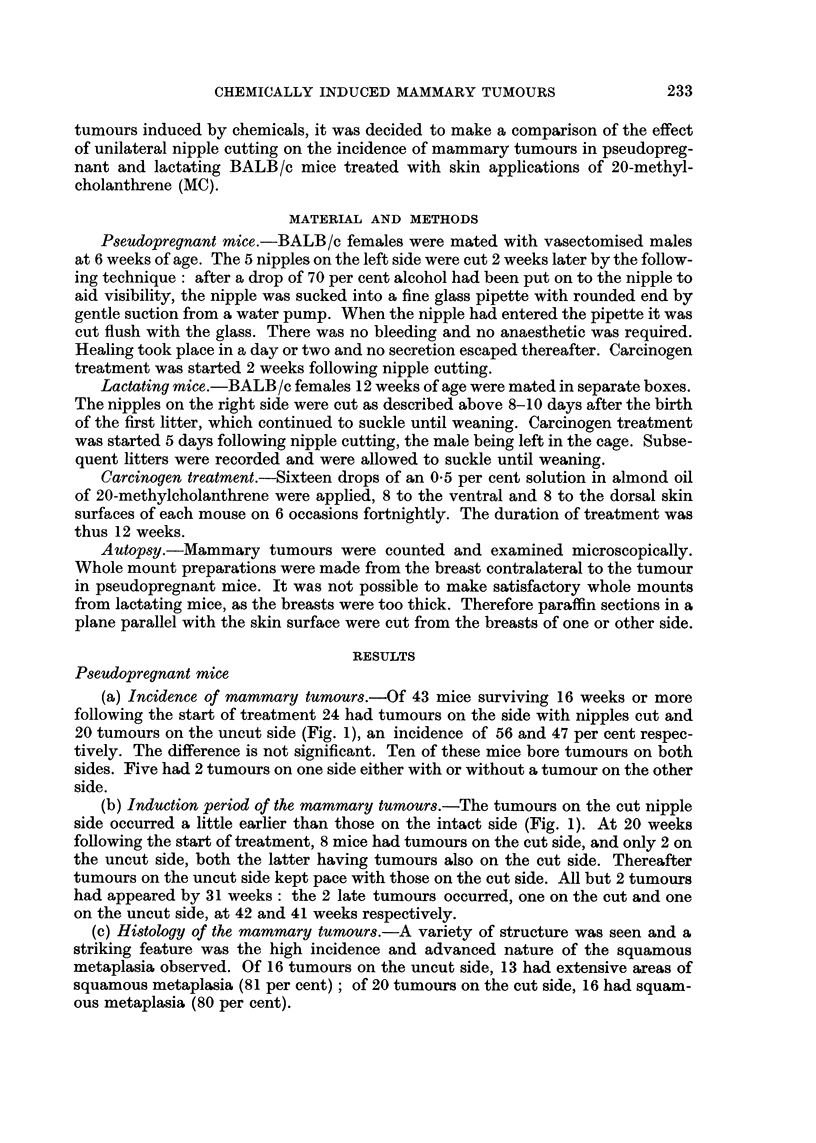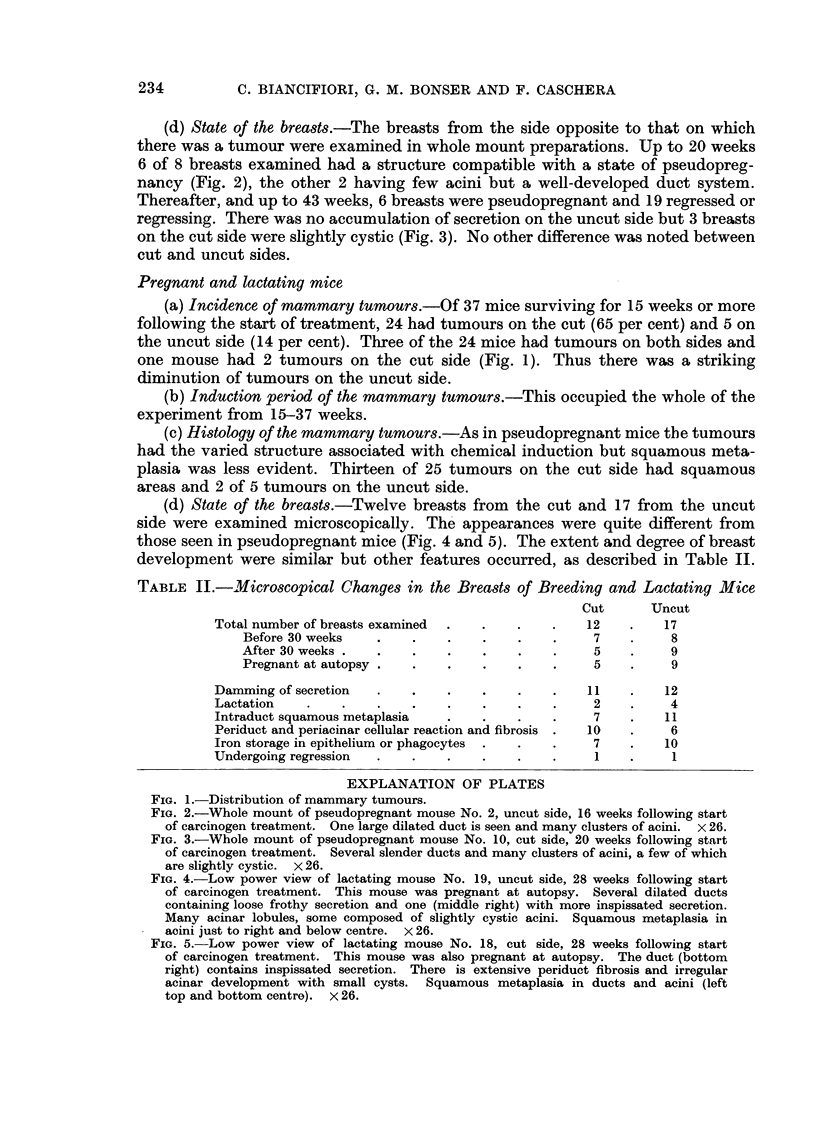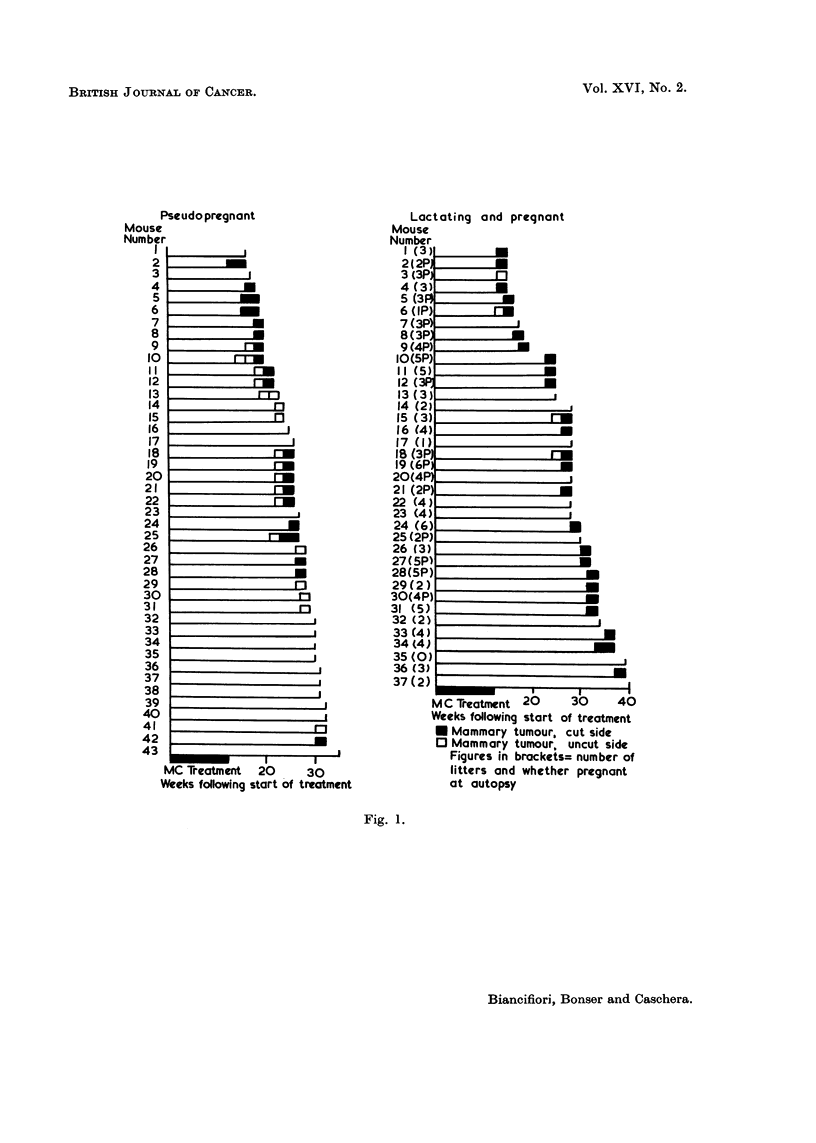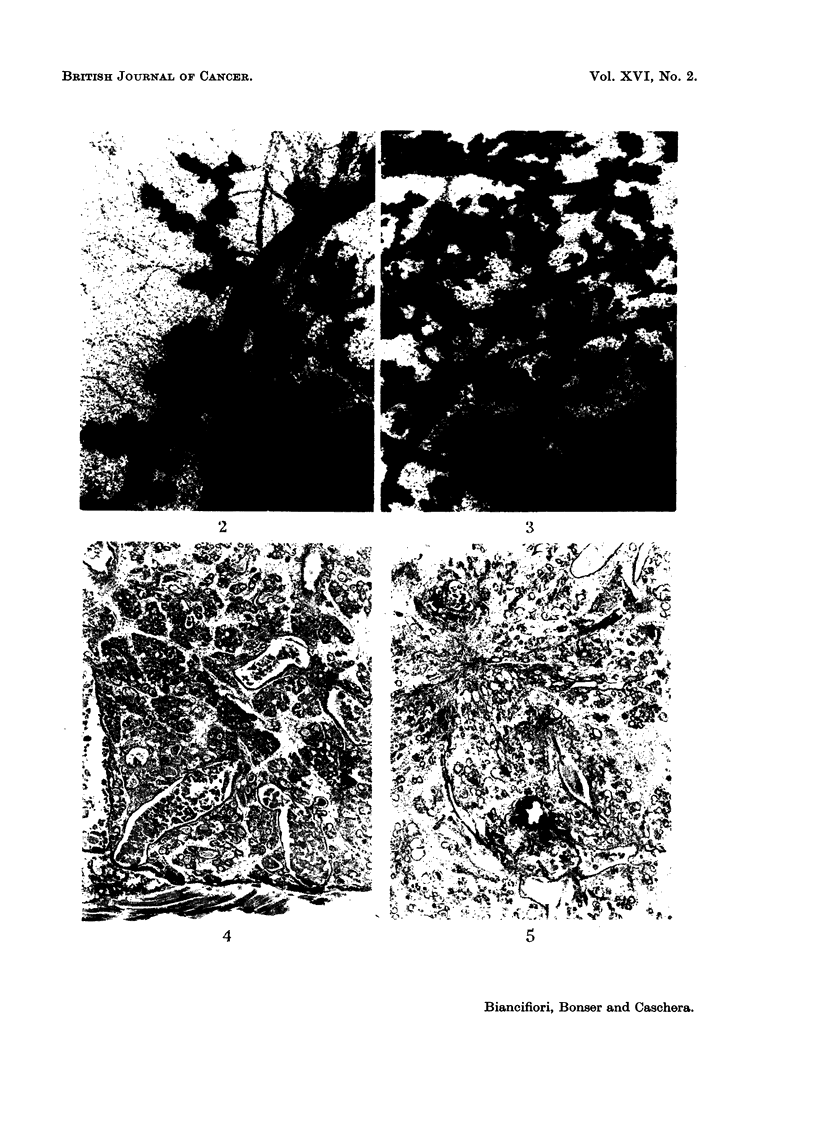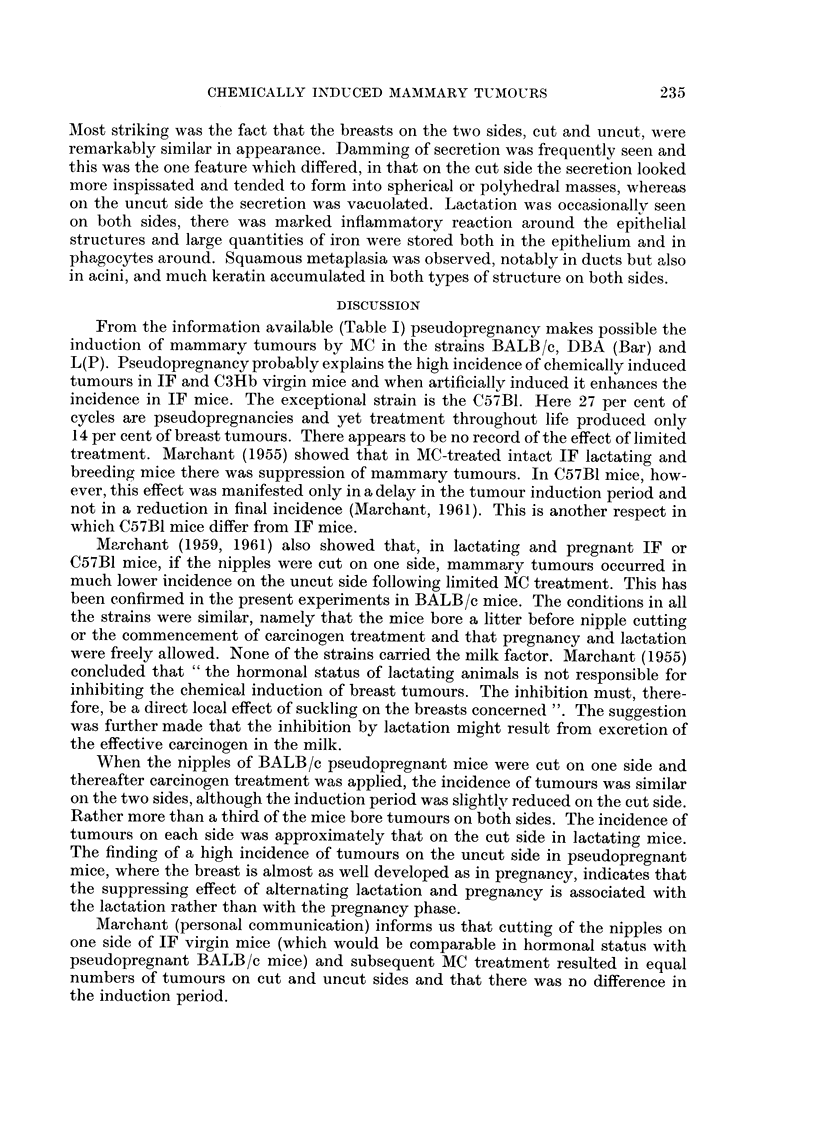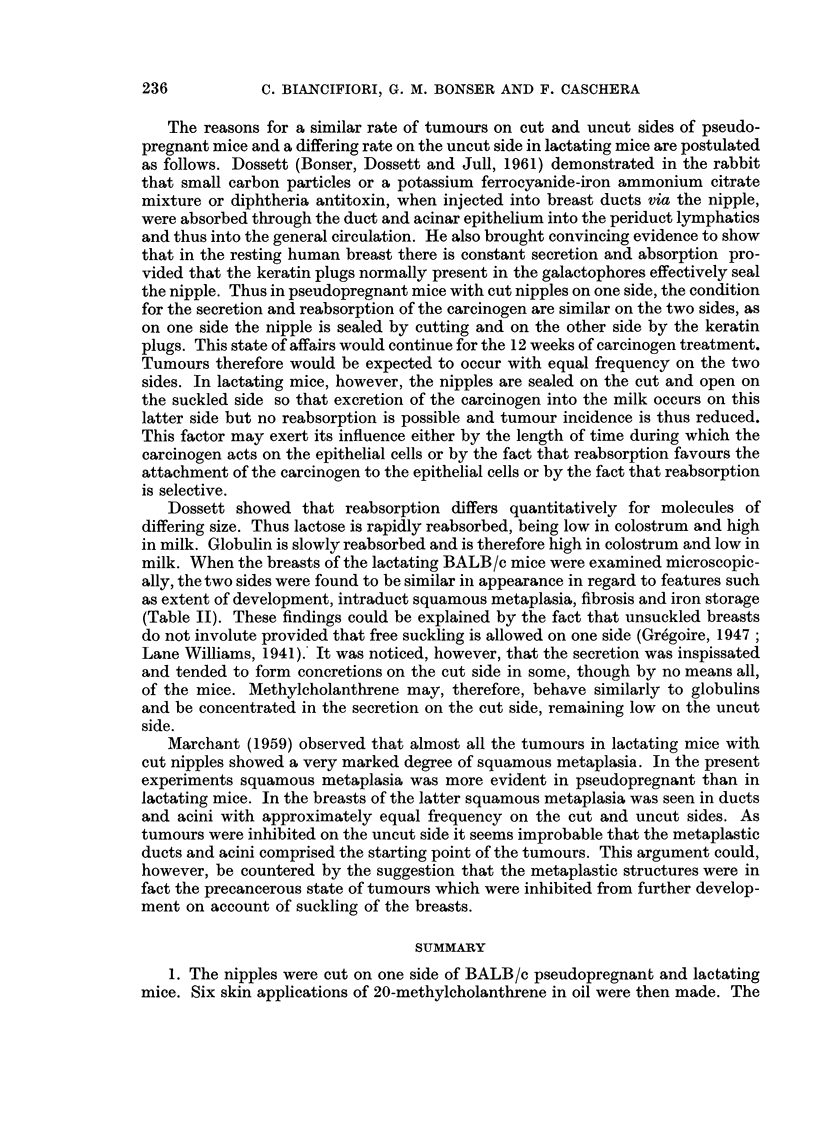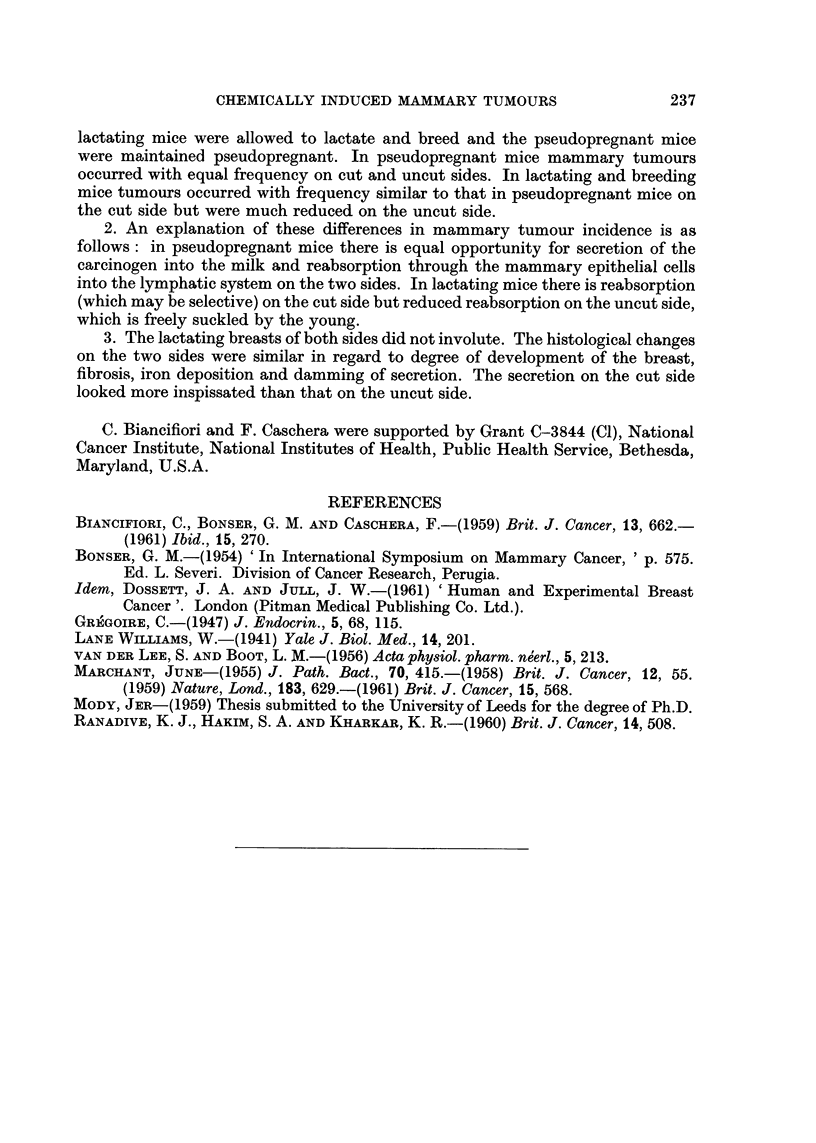# Chemically Induced Mammary Tumours Following Unilateral Excision of the Nipples in Pseudo-Pregnant and Lactating Breeding BALB/c Mice

**DOI:** 10.1038/bjc.1962.26

**Published:** 1962-06

**Authors:** C. Biancifiori, G. M. Bonser, F. Caschera

## Abstract

**Images:**


### CHEMICALLY INDUCED MAMMARY TUMOURS FOLLOWING UNILATERAL EXCISION OF THE NIPPLES IN PSEUDO-PREGNANT AND LACTATING BREEDING BALB/C MICE

C. BIANCIFIORI, G. M. BONSER AND F. CASCHERA

*From the Division of Cancer Research, University of Study, Perugia, and the Department of Experimental Pathology and Cancer Research, University of Leeds*

Received for publication March 7, 1962

FEMALE mice of strains lacking the milk factor respond in different ways to the mammary tumour inducing action of 20-methylcholanthrene. As the experiments have been done in several laboratories employing different techniques it is not always possible to make direct comparisons between the strains, but the general conclusion may be drawn that frequent pseudopregnancies greatly favour the development of chemically induced mammary tumours (Table I).

TABLE I.—*Mammary Cancer Incidence in Various Strains Following MC Treatment*

| Strain | Author | MC treatment: Limited | MC treatment: Whole experiment | Percentage incidence of mammary tumours: Virgin | Percentage incidence of mammary tumours: Pseudo-pregnant | Virgin incidence of pseudopregancy |
|---|---|---|---|---|---|---|
| BALB/c | Biancifiori *et al.* (1959) | Yes | — | 0 (36) | 44 (34) | Low (4%) |
| DBA(Bar) | Ranadive *et al.*(1960) | — | Yes | 0 (7) | 100 (8)* | — |
| L(P) | ,, ,, | — | ,, | 0 (8) | 100 (11)* | — |
| C57Bl | Marchant (1961) | — | ,, | 14 (14) | — | Medium (27%)† |
| | | Yes | — | — | 52 (21) | — |
| IF | Marchant (1958) | ,, | — | 74 (14) | 100 (9) | High ‡ |
| | Bonser (1954) | ,, | — | 44 (39) | — | — |
| C3Hb | Biancifiori *et al.* (1961) | ,, | — | 54 (36) | — | Medium (14%) |

Figures in brackets = number of mice in experiment.
* Fallopian tubes ligated.
† van der Lee and Boot (1956).
‡ Mody (1959).

Marchant (1955, 1958) showed that lactation before and pregnancy and lactation during chemical treatment by 20-methylcholanthrene greatly reduced the incidence of mammary tumours in IF mice and certain of their hybrids compared with that in virgins and pseudopregnant mice. However, the suppressing effect of lactation and pregnancy on the incidence of tumours in C57Bl mice was not observed, although there was delay in the time of appearance of the tumours (Marchant, 1961). Further, if the nipples of the lactating mice were excised on one side (Marchant, 1959, 1961) the mammary tumours were concentrated on that side in both the IF and C57Bl strains.

In view of the important effects which pseudopregnancy, lactation and pregnancy, excision of the nipples and strain exert on the incidence of mammary tumours induced by chemicals, it was decided to make a comparison of the effect of unilateral nipple cutting on the incidence of mammary tumours in pseudopregnant and lactating BALB/c mice treated with skin applications of 20-methylcholanthrene (MC).

### MATERIAL AND METHODS

*Pseudopregnant mice.*—BALB/c females were mated with vasectomised males at 6 weeks of age. The 5 nipples on the left side were cut 2 weeks later by the following technique : after a drop of 70 per cent alcohol had been put on to the nipple to aid visibility, the nipple was sucked into a fine glass pipette with rounded end by gentle suction from a water pump. When the nipple had entered the pipette it was cut flush with the glass. There was no bleeding and no anaesthetic was required. Healing took place in a day or two and no secretion escaped thereafter. Carcinogen treatment was started 2 weeks following nipple cutting.

*Lactating mice.*—BALB/c females 12 weeks of age were mated in separate boxes. The nipples on the right side were cut as described above 8–10 days after the birth of the first litter, which continued to suckle until weaning. Carcinogen treatment was started 5 days following nipple cutting, the male being left in the cage. Subsequent litters were recorded and were allowed to suckle until weaning.

*Carcinogen treatment.*—Sixteen drops of an 0·5 per cent solution in almond oil of 20-methylcholanthrene were applied, 8 to the ventral and 8 to the dorsal skin surfaces of each mouse on 6 occasions fortnightly. The duration of treatment was thus 12 weeks.

*Autopsy.*—Mammary tumours were counted and examined microscopically. Whole mount preparations were made from the breast contralateral to the tumour in pseudopregnant mice. It was not possible to make satisfactory whole mounts from lactating mice, as the breasts were too thick. Therefore paraffin sections in a plane parallel with the skin surface were cut from the breasts of one or other side.

### RESULTS

### *Pseudopregnant mice*

(a) *Incidence of mammary tumours.*—Of 43 mice surviving 16 weeks or more following the start of treatment 24 had tumours on the side with nipples cut and 20 tumours on the uncut side (Fig. 1), an incidence of 56 and 47 per cent respectively. The difference is not significant. Ten of these mice bore tumours on both sides. Five had 2 tumours on one side either with or without a tumour on the other side.

(b) *Induction period of the mammary tumours.*—The tumours on the cut nipple side occurred a little earlier than those on the intact side (Fig. 1). At 20 weeks following the start of treatment, 8 mice had tumours on the cut side, and only 2 on the uncut side, both the latter having tumours also on the cut side. Thereafter tumours on the uncut side kept pace with those on the cut side. All but 2 tumours had appeared by 31 weeks : the 2 late tumours occurred, one on the cut and one on the uncut side, at 42 and 41 weeks respectively.

(c) *Histology of the mammary tumours.*—A variety of structure was seen and a striking feature was the high incidence and advanced nature of the squamous metaplasia observed. Of 16 tumours on the uncut side, 13 had extensive areas of squamous metaplasia (81 per cent) ; of 20 tumours on the cut side, 16 had squamous metaplasia (80 per cent).

(d) *State of the breasts*.—The breasts from the side opposite to that on which there was a tumour were examined in whole mount preparations. Up to 20 weeks 6 of 8 breasts examined had a structure compatible with a state of pseudopregnancy (Fig. 2), the other 2 having few acini but a well-developed duct system. Thereafter, and up to 43 weeks, 6 breasts were pseudopregnant and 19 regressed or regressing. There was no accumulation of secretion on the uncut side but 3 breasts on the cut side were slightly cystic (Fig. 3). No other difference was noted between cut and uncut sides.

*Pregnant and lactating mice*

(a) *Incidence of mammary tumours*.—Of 37 mice surviving for 15 weeks or more following the start of treatment, 24 had tumours on the cut (65 per cent) and 5 on the uncut side (14 per cent). Three of the 24 mice had tumours on both sides and one mouse had 2 tumours on the cut side (Fig. 1). Thus there was a striking diminution of tumours on the uncut side.

(b) *Induction period of the mammary tumours*.—This occupied the whole of the experiment from 15–37 weeks.

(c) *Histology of the mammary tumours*.—As in pseudopregnant mice the tumours had the varied structure associated with chemical induction but squamous metaplasia was less evident. Thirteen of 25 tumours on the cut side had squamous areas and 2 of 5 tumours on the uncut side.

(d) *State of the breasts*.—Twelve breasts from the cut and 17 from the uncut side were examined microscopically. The appearances were quite different from those seen in pseudopregnant mice (Fig. 4 and 5). The extent and degree of breast development were similar but other features occurred, as described in Table II.

TABLE II.—*Microscopical Changes in the Breasts of Breeding and Lactating Mice*

| | Cut | Uncut |
|---|---|---|
| Total number of breasts examined | 12 | 17 |
| Before 30 weeks | 7 | 8 |
| After 30 weeks | 5 | 9 |
| Pregnant at autopsy | 5 | 9 |
| Damming of secretion | 11 | 12 |
| Lactation | 2 | 4 |
| Intraduct squamous metaplasia | 7 | 11 |
| Periduct and periacinar cellular reaction and fibrosis | 10 | 6 |
| Iron storage in epithelium or phagocytes | 7 | 10 |
| Undergoing regression | 1 | 1 |

EXPLANATION OF PLATES

FIG. 1.—Distribution of mammary tumours.

FIG. 2.—Whole mount of pseudopregnant mouse No. 2, uncut side, 16 weeks following start of carcinogen treatment. One large dilated duct is seen and many clusters of acini. ×26.

FIG. 3.—Whole mount of pseudopregnant mouse No. 10, cut side, 20 weeks following start of carcinogen treatment. Several slender ducts and many clusters of acini, a few of which are slightly cystic. ×26.

FIG. 4.—Low power view of lactating mouse No. 19, uncut side, 28 weeks following start of carcinogen treatment. This mouse was pregnant at autopsy. Several dilated ducts containing loose frothy secretion and one (middle right) with more inspissated secretion. Many acinar lobules, some composed of slightly cystic acini. Squamous metaplasia in acini just to right and below centre. ×26.

FIG. 5.—Low power view of lactating mouse No. 18, cut side, 28 weeks following start of carcinogen treatment. This mouse was also pregnant at autopsy. The duct (bottom right) contains inspissated secretion. There is extensive periduct fibrosis and irregular acinar development with small cysts. Squamous metaplasia in ducts and acini (left top and bottom centre). ×26.

Pseudopregnant
Mouse Number
MC Treatment
20
30
Weeks following start of treatment
Lactating and pregnant
Mouse Number
1 (3)
2 (2P)
3 (3P)
4 (3)
5 (3P)
6 (1P)
7 (3P)
8 (3P)
9 (4P)
10 (5P)
11 (5)
12 (3P)
13 (3)
14 (2)
15 (3)
16 (4)
17 (1)
18 (3P)
19 (6P)
20 (4P)
21 (2P)
22 (4)
23 (4)
24 (6)
25 (2P)
26 (3)
27 (5P)
28 (5P)
29 (2)
30 (4P)
31 (5)
32 (2)
33 (4)
34 (4)
35 (0)
36 (3)
37 (2)
MC Treatment
20
30
40
Weeks following start of treatment
Mammary tumour, cut side
Mammary tumour, uncut side
Figures in brackets= number of litters and whether pregnant at autopsy

Fig. 1.

2

3

4

5

Biancifiori, Bonser and Caschera.

Most striking was the fact that the breasts on the two sides, cut and uncut, were remarkably similar in appearance. Damming of secretion was frequently seen and this was the one feature which differed, in that on the cut side the secretion looked more inspissated and tended to form into spherical or polyhedral masses, whereas on the uncut side the secretion was vacuolated. Lactation was occasionally seen on both sides, there was marked inflammatory reaction around the epithelial structures and large quantities of iron were stored both in the epithelium and in phagocytes around. Squamous metaplasia was observed, notably in ducts but also in acini, and much keratin accumulated in both types of structure on both sides.

### DISCUSSION

From the information available (Table I) pseudopregnancy makes possible the induction of mammary tumours by MC in the strains BALB/c, DBA (Bar) and L(P). Pseudopregnancy probably explains the high incidence of chemically induced tumours in IF and C3Hb virgin mice and when artificially induced it enhances the incidence in IF mice. The exceptional strain is the C57Bl. Here 27 per cent of cycles are pseudopregnancies and yet treatment throughout life produced only 14 per cent of breast tumours. There appears to be no record of the effect of limited treatment. Marchant (1955) showed that in MC-treated intact IF lactating and breeding mice there was suppression of mammary tumours. In C57Bl mice, however, this effect was manifested only in a delay in the tumour induction period and not in a reduction in final incidence (Marchant, 1961). This is another respect in which C57Bl mice differ from IF mice.

Marchant (1959, 1961) also showed that, in lactating and pregnant IF or C57Bl mice, if the nipples were cut on one side, mammary tumours occurred in much lower incidence on the uncut side following limited MC treatment. This has been confirmed in the present experiments in BALB/c mice. The conditions in all the strains were similar, namely that the mice bore a litter before nipple cutting or the commencement of carcinogen treatment and that pregnancy and lactation were freely allowed. None of the strains carried the milk factor. Marchant (1955) concluded that " the hormonal status of lactating animals is not responsible for inhibiting the chemical induction of breast tumours. The inhibition must, therefore, be a direct local effect of suckling on the breasts concerned ". The suggestion was further made that the inhibition by lactation might result from excretion of the effective carcinogen in the milk.

When the nipples of BALB/c pseudopregnant mice were cut on one side and thereafter carcinogen treatment was applied, the incidence of tumours was similar on the two sides, although the induction period was slightly reduced on the cut side. Rather more than a third of the mice bore tumours on both sides. The incidence of tumours on each side was approximately that on the cut side in lactating mice. The finding of a high incidence of tumours on the uncut side in pseudopregnant mice, where the breast is almost as well developed as in pregnancy, indicates that the suppressing effect of alternating lactation and pregnancy is associated with the lactation rather than with the pregnancy phase.

Marchant (personal communication) informs us that cutting of the nipples on one side of IF virgin mice (which would be comparable in hormonal status with pseudopregnant BALB/c mice) and subsequent MC treatment resulted in equal numbers of tumours on cut and uncut sides and that there was no difference in the induction period.

The reasons for a similar rate of tumours on cut and uncut sides of pseudopregnant mice and a differing rate on the uncut side in lactating mice are postulated as follows. Dossett (Bonser, Dossett and Jull, 1961) demonstrated in the rabbit that small carbon particles or a potassium ferrocyanide-iron ammonium citrate mixture or diphtheria antitoxin, when injected into breast ducts *via* the nipple, were absorbed through the duct and acinar epithelium into the periduct lymphatics and thus into the general circulation. He also brought convincing evidence to show that in the resting human breast there is constant secretion and absorption provided that the keratin plugs normally present in the galactophores effectively seal the nipple. Thus in pseudopregnant mice with cut nipples on one side, the condition for the secretion and reabsorption of the carcinogen are similar on the two sides, as on one side the nipple is sealed by cutting and on the other side by the keratin plugs. This state of affairs would continue for the 12 weeks of carcinogen treatment. Tumours therefore would be expected to occur with equal frequency on the two sides. In lactating mice, however, the nipples are sealed on the cut and open on the suckled side so that excretion of the carcinogen into the milk occurs on this latter side but no reabsorption is possible and tumour incidence is thus reduced. This factor may exert its influence either by the length of time during which the carcinogen acts on the epithelial cells or by the fact that reabsorption favours the attachment of the carcinogen to the epithelial cells or by the fact that reabsorption is selective.

Dossett showed that reabsorption differs quantitatively for molecules of differing size. Thus lactose is rapidly reabsorbed, being low in colostrum and high in milk. Globulin is slowly reabsorbed and is therefore high in colostrum and low in milk. When the breasts of the lactating BALB/c mice were examined microscopically, the two sides were found to be similar in appearance in regard to features such as extent of development, intraduct squamous metaplasia, fibrosis and iron storage (Table II). These findings could be explained by the fact that unsuckled breasts do not involute provided that free suckling is allowed on one side (Grégoire, 1947 ; Lane Williams, 1941). It was noticed, however, that the secretion was inspissated and tended to form concretions on the cut side in some, though by no means all, of the mice. Methylcholanthrene may, therefore, behave similarly to globulins and be concentrated in the secretion on the cut side, remaining low on the uncut side.

Marchant (1959) observed that almost all the tumours in lactating mice with cut nipples showed a very marked degree of squamous metaplasia. In the present experiments squamous metaplasia was more evident in pseudopregnant than in lactating mice. In the breasts of the latter squamous metaplasia was seen in ducts and acini with approximately equal frequency on the cut and uncut sides. As tumours were inhibited on the uncut side it seems improbable that the metaplastic ducts and acini comprised the starting point of the tumours. This argument could, however, be countered by the suggestion that the metaplastic structures were in fact the precancerous state of tumours which were inhibited from further development on account of suckling of the breasts.

### SUMMARY

1. The nipples were cut on one side of BALB/c pseudopregnant and lactating mice. Six skin applications of 20-methylcholanthrene in oil were then made. The lactating mice were allowed to lactate and breed and the pseudopregnant mice were maintained pseudopregnant. In pseudopregnant mice mammary tumours occurred with equal frequency on cut and uncut sides. In lactating and breeding mice tumours occurred with frequency similar to that in pseudopregnant mice on the cut side but were much reduced on the uncut side.

2. An explanation of these differences in mammary tumour incidence is as follows : in pseudopregnant mice there is equal opportunity for secretion of the carcinogen into the milk and reabsorption through the mammary epithelial cells into the lymphatic system on the two sides. In lactating mice there is reabsorption (which may be selective) on the cut side but reduced reabsorption on the uncut side, which is freely suckled by the young.

3. The lactating breasts of both sides did not involute. The histological changes on the two sides were similar in regard to degree of development of the breast, fibrosis, iron deposition and damming of secretion. The secretion on the cut side looked more inspissated than that on the uncut side.

C. Biancifiori and F. Caschera were supported by Grant C–3844 (Cl), National Cancer Institute, National Institutes of Health, Public Health Service, Bethesda, Maryland, U.S.A.

### REFERENCES

Biancifiori, C., Bonser, G. M. and Caschera, F.—(1959) *Brit. J. Cancer*, **13**, 662.—(1961) *Ibid.*, **15**, 270.

Bonser, G. M.—(1954) ' In International Symposium on Mammary Cancer, ' p. 575. Ed. L. Severi. Division of Cancer Research, Perugia.

*Idem*, Dossett, J. A. and Jull, J. W.—(1961) ' Human and Experimental Breast Cancer '. London (Pitman Medical Publishing Co. Ltd.).

Grégoire, C.—(1947) *J. Endocrin.*, **5**, 68, 115.

Lane Williams, W.—(1941) *Yale J. Biol. Med.*, **14**, 201.

van der Lee, S. and Boot, L. M.—(1956) *Acta physiol. pharm. néerl.*, **5**, 213.

Marchant, June—(1955) *J. Path. Bact.*, **70**, 415.—(1958) *Brit. J. Cancer*, **12**, 55. (1959) *Nature, Lond.*, **183**, 629.—(1961) *Brit. J. Cancer*, **15**, 568.

Mody, Jer—(1959) Thesis submitted to the University of Leeds for the degree of Ph.D.

Ranadive, K. J., Hakim, S. A. and Kharkar, K. R.—(1960) *Brit. J. Cancer*, **14**, 508.